# Global health research, partnership, and equity: no more business-as-usual

**DOI:** 10.1186/1472-698X-11-S2-S1

**Published:** 2011-11-08

**Authors:** Christina Zarowsky

**Affiliations:** 1 University of the Western Cape, School of Public Health, Bellville, South Africa

## 

The papers in this important collection reflect a mature and confident way of doing global health research which is anything but business-as-usual. In the context of increasing competition for individual or institutional “leadership” of the field (and business) of global health, these contributors instead speak of active and sustained collaboration -- listening, responsiveness, flexibility, willingness and capacity to follow as well as to lead -- in learning what to transform or sustain, and how, in order to move towards greater equity in both health and health research. Each paper and the collection as a whole is an important contribution to the evidence base for a range of issues from maternal health, HIV and access to services, to chronic disease, health system strengthening, occupational health, ecosystemic approaches to health, and social inclusion, exclusion, and neglect. In addition, they challenge conventional models of research focused on narrowly defined research questions and a narrow range of pre-specified research methods, documenting instead how both the research questions and the methods most appropriate to address them change over time. Finally, they challenge both the idea of “pure” science undertaken by independent researchers on behalf of science and specific communities, and the conventional wisdom that North-South and research-research user-community partnerships are necessarily either North and researcher-driven, or scientifically dubious. The papers are, on the whole, circumspect in their claims, and honest about the limitations and frustrations facing research-based teams seeking to challenge or transform entrenched socio-political hierarchies and inequities. This is an invigorating and informative collection of good science, good partnership, and important results.

Some of the papers are more transparent than others about the challenges of such a counter-cultural approach to “excellence” and “impact” of research. All of the contributions reflect on the importance of context, time to develop trust and relationships, the centrality of distributive justice and of developing processes for conducting complex research and advocacy ethically, the powerful contributions of research excellence to creating both social spaces and sound interventions to improve health equity, and the relevance as well as changing balance over time of different conceptual and methodological approaches. What is less clear is the additional intellectual, administrative, and emotional work that is needed to achieve the remarkable results described in the papers, and the costs this carries relative to the still-dominant approaches where an outside “expert” comes in for short periods of fieldwork, arranges the collection of data, analyses it, and writes it up for publication – often without “southern” (let alone service provider or community) co-authors. As observed by MacFarlane et al (2008:389), “Of 434 papers ever published in the PubMed database (searched in May 2008) by authors with “global health” and (“university” or “institute” or “college” or “school”) in their affiliation, 87% were from North American institutions” [1].

The commitment to genuine partnership, relevance and strengthening of local capacities to identify, analyze and transform the complex conditions that create and sustain inequity is not compatible with funding or academic advancement models that privilege fully pre-determined research questions and instruments, short timelines requiring rapid implementation of interventions, ready attribution of clear outcomes to specific funders, and rapid publication of numerous scientific papers (which are preferably short, not too complex, and fluently written, usually in English). Collaborative approaches in contrast require (re)negotiation of everything from objectives to governance, and sensitivity to and respect for the various and at times diverging agendas and constraints of various “stakeholders”. This sensitivity and respect almost always require tolerance for disagreement, taking time to build and maintain trust and a common vision, attention to protocol and the details of who participates in both financial and scientific decision making. It usually means that progress, and publication, is slower than researchers (and funders) would like. It sometimes means that manuscripts are less elegant or incisive than an experienced researcher and author would like. Rarely, it may mean that findings are not published at all, and that researchers and other partners must decide whether both the public good and the welfare of the study participants and partners are better served by disclosing, delaying or not disclosing certain findings. Read these manuscripts, and between the lines, see how these challenges have been encountered and usually surmounted by an extraordinary set of research teams. Then consider joining them, if you haven’t already.

It is now two years since I left IDRC and joined the School of Public Health at the University of the Western Cape. Many – in fact, all – of my new colleagues are involved in the kind of research collaborations described in this collection. My respect for them and their work, and for the contributors to this collection, is even greater now that I have some sense of what “research capacity strengthening” and “partnership” mean from the other side of the funder’s table and the other side of the equator. And my appreciation for the flexible and supportive approach to research funding demonstrated by IDRC and a handful of other funders has grown apace.

This collection, and other work in this spirit, is a challenge to the academy and the “peers” who review and adjudicate our work, to research funders, to scientific journals, and to policy makers and other potential “research users” who may be afraid to risk the transparency of research committed to excellence and to transforming the world. I hope they will take up the challenge.

## Competing interests

The author's salary is partially supported through research grants from IDRC Canada. The opinions expressed here are solely those of the author.
